# A comparative, randomized clinical trial of artemisinin/naphtoquine twice daily one day *versus *artemether/lumefantrine six doses regimen in children and adults with uncomplicated falciparum malaria in Côte d'Ivoire

**DOI:** 10.1186/1475-2875-8-148

**Published:** 2009-07-03

**Authors:** Offianan A Toure, Louis K Penali, Jean-Didier Yapi, Berenger A Ako, Walamtchin Toure, Kali Djerea, Genevieve O Gomez, Oyewole Makaila

**Affiliations:** 1 Malariology department, Institut Pasteur de Côte d'Ivoire, PO Box: 490 Abidjan 01, Côte d'Ivoire; 2 Hospital of Bonoua, Côte d'Ivoire; 3 Health Center of Anonkoua-Koute, Abidjan, Côte d'Ivoire

## Abstract

**Background:**

Drug resistance in *Plasmodium falciparum *poses a major threat to malaria control. Combination anti-malarial therapy, including artemisinins, has been advocated to improve efficacy and limit the spread of resistance. The fixed combination of oral artemether-lumefantrine (AL) is highly effective and well-tolerated. Artemisinin/naphtoquine (AN) is a fixed-dose ACT that has recently become available in Africa.

The objectives of the study were to compare the efficacy and safety of AN and AL for the treatment of uncomplicated *falciparum *malaria in a high transmission-intensity site in Ivory Coast.

**Methods:**

We enrolled 122 participants aged 6 months or more with uncomplicated *falciparum *malaria. Participants were randomized to receive either artemisinin/naphtoquine or artemether/lumefantrine with variable dose according to their weight. Primary endpoints were the risks of treatment failure within 28 days, either unadjusted or adjusted by genotyping to distinguish recrudescence from new infection.

**Results:**

Among 125 participants enrolled, 123 (98.4%) completed follow-up. Clinical evaluation of the 123 participants showed that cumulative PCR-uncorrected cure rate on day 28 was 100% for artemisinin/naphtoquine and 98.4% for artemether/lumefantrine. Both artemisinin-based combinations effected rapid fever and parasite clearance.

**Interpretation:**

These data suggest that Arco^® ^could prove to be suitable for use as combination antimalarial therapy. Meanwhile, pharmacokinetic studies and further efficacy assessment should be conducted before its widespread use can be supported.

## Background

Malaria remains a leading cause of morbidity and mortality in African children [1]. The basis for malaria control throughout sub-Saharan Africa is an appropriate case management, focusing on prompt treatment with effective anti-malarial drugs [2]. *Plasmodium falciparum *resistance to affordable anti-malarial drugs (chloroquine and sulphadoxine-pyrimethamine) has reached high levels and noticeably hampered malaria control efforts in the region [3].

Artemisinin-based combination therapy (ACT) has proved effectiveness against malaria in Asia and Africa [4,5], and most countries in Africa have now changed their national treatment policy to incorporate artemisinin-based combination regimens as first-line treatment for uncomplicated malaria [6].

Because artemisinin derivatives are now the first-line treatment for multidrug-resistant *falciparum *malaria in many tropical countries, appearance of artemisinin-resistant *Plasmodium falciparum *would have serious implications.

The development of suitable combinations of an artemisinin compound with a second drug is therefore a priority.

The choice of a suitable partner for the artemisinins has, however, been problematic.

Candidates are being developed, including lumefantrine, amodiaquine, mefloquine piperaquine, pyronaridine, and naphthoquinone.

Naphtoquine is a member of the 4-aminoquinoline group that includes chloroquine.

Detailed *in vivo *and *in vitro *data about naphtoquine are not available apart from one study published in Chinese which showed that naphtoquine is proved to be effective and well tolerated. During this study, the average fever-subsidence time and the parasite-clearance time of naphtoquine at single 24-hour dosage of 1,000 mg were longer than those of mefloquine and artesunate, the 28-day curative ratio of naphtoquine was higher than that of mefloquine and artesunate [7].

The fixed combination of artemether/lumefantrine (AL) has been used as first-line treatment for uncomplicated *falciparum *malaria in several African countries [8].

The safety of AL has been extensively reviewed [9] and several trials in Africa and all of them, in both children and adults, have demonstrated its efficacy [10,11]. AL exists as a fixed tablet formulation and it has been registered in a large number of countries under the names of Coartem^®^or Riamet^®^. The fixed tablet formulation helps to overcome problems of compliance associated with non-coformulated combinations.

Artemisinin/naphtoquine is a fixed-dose ACT that has recently become available in Africa. In the only published study evaluating Naphtoquine, this drug was highly efficacious with a good safety and tolerability profile.

However, the epidemiology of malaria and patterns of antimalarial drug used are quite different in Africa than in Asia [12,13].

To compare the performance of AN with Artemether/lumefantrine in Ivory Coast, we conducted a randomized clinical trial comparing efficacy and safety of AN and AL for the treatment of uncomplicated *falciparum *malaria in Anonkoua-koute, an area of extremely high transmission intensity.

## Methods

### Study design and study site

The study was conducted from November 2006 to January 2007 at the end of the rainy season, in the primary health care center of Anonkoua-kouté, 10 kms from Abidjan. This health care center is located in a crowded sub-urban area with holoendemic *P. falciparum *transmission.

This trial was a randomized single-blinded clinical trial.

### Participants

Inclusion criteria were the following: (1) six months old or more; (2) *P. falciparum *monoinfection with parasitaemia level of 2,000 – 200,000 asexual parasites/μl of blood; (3) axillary temperature equal or over 37.5°C at the time of enrolment or history of fever during the preceding 24 h; (4) no history of serious side effects to study medications; (5) no evidence of concomitant febrile illness; (6) provision of written informed consent by the participant or by a parent or guardian (for children).

Exclusion criteria were as follows: (1) symptoms and/or signs of severe malaria; (2) any danger sign (persistent vomiting; inability to sit, stand, drink or breastfeed; (3) recent history of convulsions and/or lethargy or otherwise impaired consciousness; (4) haemoglobin concentration ≤ 6 mg/dl; (5) serious underlying disease or known allergy to the study drugs.

Participants were also excluded after randomization, if they repeatedly vomited their first dose of study medications.

The sample size for the study was calculated based on the assumptions that day 28 PCR-corrected cure rates for the ACTs will not be less than 95%, and that the cure rate for either AN or AL will not be less than 85%. At 95% confidence interval, 80% power, and a sample ratio of 1:1, the required sample per arm was 49. Allowing for a dropout rate of 10%, a minimum of 55 participants were to be recruited for each arm of the study.

### Treatments and randomization

Participants recruited into the study were allocated to two treatment groups using a computer generated random list based on a simple random selection procedure without the use of blocking or stratification by an off-site investigator.

Sequentially numbered, sealed envelopes containing the treatment group assignments were prepared from the randomization list.

The study clinical investigators assigned treatment numbers sequentially and a third party investigator who is an appropriately qualified member of the study site, allocated treatment by opening the envelope corresponding to the treatment number. The randomization codes were secured in a locked cabinet accessible only by the third party. Participants were enrolled by the study physicians, and treatments were assigned and administered by the third party.

Only the third party was aware of treatment assignments. All other study personnel, including the study physicians and laboratory personnel involved in assessing outcomes, were blinded to the treatment assignments. Participants were not informed of their treatment regimen.

Study treatment was started on the day of randomization (day 0) and completed by day 2 according to the combination received.

The study drug Arco^® ^(AN) was provided in blister packs by Kunming Pharmaceutical Corp. (China) The active ingredients are artemisinin and naphtoquine. Each tablet contains 125 mg of artemisinin and 50 mg of naphtoquine.

The comparator drug Coartem^® ^(AL) was provided by Novartis SA (swiss) in blisters packs. The active ingredients are artemether 20 mg and lumefantrine 120 mg.

Administration of treatment was as described below.

The dosing for artemisinin/naphtoquine (AN) was 1/2 crushed tablet for participants weighing between 6 kg and 10 kg; one crushed tablet for participants weighing between 10 and 15 kg; two crushed tablets for participants weighing between 15 and 25 kg; three tablets for participants weighing between 25 and 35 kg, and four tablets for participants whose weight was at least equal to 35 kg. All tablets were given twice on a single day; the second dose being given eight hours after the first dose.

For artemether/lumefantrine (AL), the dosing was 1 crushed tablet for participants weighing between 5 and 15 kg; two crushed tablets for participants weighing between 15 and 25 kg; three tablets for participants weighing between 25 and 35 kg; four tablets for participants weighing between whose weight was at least equal to 35 kg. All tablets were given twice a day for three days; the second dose was given eight hours after the first one and the third dose 24 hours after the first one or 16 hours after the second one, the following doses given every 12 hours.

For AN, the morning doses were directly observed, while the evening dose was given to the participants to be taken at home. For AL, the morning doses were also directly observed over the three days of treatment, while the evening doses were given to participants to be taken at home. The empty sachets were returned to the study site as evidence of taking the drug. Paracetamol tablets (three doses per day for two days) were provided, to be taken if needed. Participants who were absent for follow-up were visited at home on the same day.

All tablets were either swallowed whole or crashed with water.

In the case a participant vomits the first dose (D0) within 30 minutes of drug administration; a repeat full dose was given. If this participant also vomits this repeat dose or any subsequent dosing, he was not re-dosed and was withdrawn from the study. A participant who was withdrawn due to vomiting received rescue medication according to local practices.

### Follow-up procedures

Enrolled participants were given an identity code and were assigned to receive either artemisinin/naphtoquine (125/50 mg tablet) twice on a single day (Day 0) or artemether/lumefantrine (20/120 mg tablet) twice a day for three days (Day 0, 1 and 2). At enrolment, we asked participants, children's parents or guardians about prior anti-malarial therapy, use of other medications, and presence of common symptoms. Axillary temperature and weight were recorded, and a physical examination was performed. We also collected blood by fingerprick for thick and thin blood smears, and for storage on filter paper for PCR analysis.

Participants were recommended to return to study site for follow-up visit on days 1, 2, 3, 7, 14, 21 and 28, and any other day, if they felt ill. Follow-up evaluation consisted in a standardized history and physical examination. If participants did not return for follow-up, they were visited at home.

Participants were excluded after enrolment if any of the following occurred: (1) use of antimalarial drugs outside of the study protocol; (2) parasitaemia in the presence of a concomitant febrile illness; (3) withdrawal of consent; (4) loss to follow-up, (5) protocol violation, or (6) death due to a non-malaria illness.

### Outcome

Treatment outcomes were classified according to 2003 World Health Organization (WHO) guidelines as early treatment failure (ETF: danger signs or complicated malaria or failure to adequately respond to therapy days 0–3); late clinical failure (LCF: danger signs or complicated malaria or fever and parasitemia on days 4–28 without previously meeting criteria for ETF); late parasitological failure (LPF: asymptomatic parasitemia on day 28 without previously meeting criteria for ETF or LCF); and adequate clinical and parasitological response (ACPR: absence of parasitemia on day 28 without previously meeting criteria for ETF, LCF, or LPF) [WHO assessment].

The overall rate of treatment failure (Total Treatment Failure TTF) was computed as if the participant had an ETF, LCF or a LPF. Only parasitaemia confirmed by PCR as recrudescence was considered a treatment failure. Participants were also considered treatment failures if they received rescue treatment on or before day 28.

### Adverse event

An adverse event is defined as any unfavourable and unintended sign, symptom, syndrome, or illness that develops or worsens during the period of observation in the study.

Clinically relevant abnormal results of diagnostic procedures including abnormal laboratory findings which was considered by the investigator to be detrimental was recorded as adverse events whether or not they have a causal relationship with the study drug.

All observed adverse events were monitored actively and passively from the time the participant has taken one dose of study treatment through last visit, and were recorded on the Case Report Form (CRF) according to Good Clinical Practice (GCP) and ICH guidelines.

### Laboratory investigation

Thick and thin blood smears were prepared and stained in 10% Giemsa solution for 30 minutes. The smears were read to 100 fields with quantification of *P. falciparum *asexual parasitaemia on the thick smear. Parasites were enumerated using thick film, as described by Shute [14]. The parasite density (per μl of blood) was calculated, assuming a normal leucocyte level of 8,000/μl. The thin film was used to speciate the parasites.

A smear was considered negative if no parasite were seen after review of 100 high-power fields. We also assessed gametocytemia from thick blood smears. Thin blood smears were reviewed for non-*falciparum *infections. A second microscopist, who was unaware of the results of the first reading, re-read all slides. A third microscopist unaware of the first two readings resolved discrepant slides.

A complete blood count plus serum alanine aminotransferase (ALT), serum asparate aminotransferase, serum total bilirubin and serum creatinine assessments were performed on the total participants at baseline and seven days after initiation of treatment. Tests were also performed if clinically indicated or if a significant abnormality was detected on day 7. Molecular genotyping techniques were used to distinguish recrudescence from new infection for all participants failing therapy after day 7. Briefly, filter paper blood samples collected on the day of enrolment and on the day of failure were analyzed for polymorphisms in the genes for merozoite surface protein-1 (*msp*-1) and merozoite surface protein-2 (*msp*-2) using nested-PCR as previously described [15].

### Management of recrudescent infections

Participants with uncomplicated recrudescent infections were re-treated with quinine, 24 mg/kg/day for 5 days. However, their response to repeat therapy was not assessed.

### Statistical analysis

Data generated were recorded in a log book and individual participants case record files. Data were entered and analysed with EPI-Info version 6.4. Analysis of treatment outcome was per protocol, which only included participants with treatment outcomes. Frequencies were compared by chi-squared tests and Fisher exact tests, and continuous variables by Student's t-tests, Mann-Whitney U-tests, analysis of variance or Kruskal-Wallis tests as applicable.

All the clinical and laboratory data collected were subjected to quality control. The study was carried out according to standard operating procedures following the guidelines of good clinical practice.

### Ethics

The study was approved by our Ethical Committee. This work was conducted in compliance with ICH-GCP guidelines. Patient's informed consent was obtained according to the ethical principles stated in the declaration of Helsinki 2000 version (amended in Tokyo 2004), the applicable guidelines for ICH-GCP. The applicable laws and regulations of Ivory Coast Informed consent document was used to explain the risks and benefits of study participation to the participant if adults or parent or guardian of children participants in simple terms before the participant was entered into the study. The informed consent document contains a statement that the consent is freely given, parent or guardian of the participant was aware of the risks and benefits of entering the study, and the participant is free to withdraw from the study at any time. Written consent was provided by participant or parent or guardian. If the participant or parent or guardian was unable to write, witnessed consent was permitted.

## Results

### Trial profile

A total of 524 participants were screened for study eligibility and 125 who fulfilled the inclusion criteria were enrolled in the study (63 in AN arm and 62 in AL arm). All participants gave their written informed consent before participating in the study.

**Figure 1:**
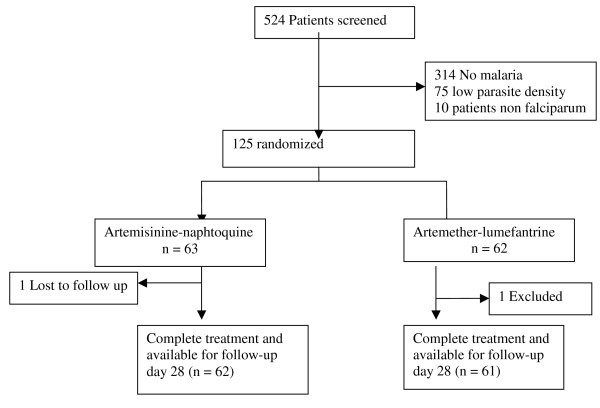
**A total of 123 participants over 125 complied freely with the study protocol up to the time of a new malaria episode or follow-up day 28 and had adequate data for the analysis of the endpoints**.

A total of 123 participants over 125 complied freely with the study protocol up to the time of a new malaria episode or follow-up day 28 and had adequate data for the analysis of the end-points (Figure 1).

Two participants were lost to follow-up: one child in the AN group and one in the AL group. Table 1 shows the baseline characteristics of the eligible participants. The two treatment groups were comparable in all characteristics.

**Table 1:** Demographic, clinical, and parasitological characteristics of participants on day of recruitment

Male/Female), range	27/35	29/32**
Mean weight (kg.), range	41.52 (11–77)	41.40 (10–82) **
GM parasite count (μl)a, range	10 186.13 (2000–102420)	11 081.8 (2000–99120) **
Mean haemoglobin (g/dl)	11.02 (6.56–14.7)	10.9 (7.4–15.9) **

AN (n = 62) AL (n = 61)

^a ^Geometric mean parasite count

* p = 0.01 **p > 0.05

### Parasites and fever clearance

**Figure 2:**
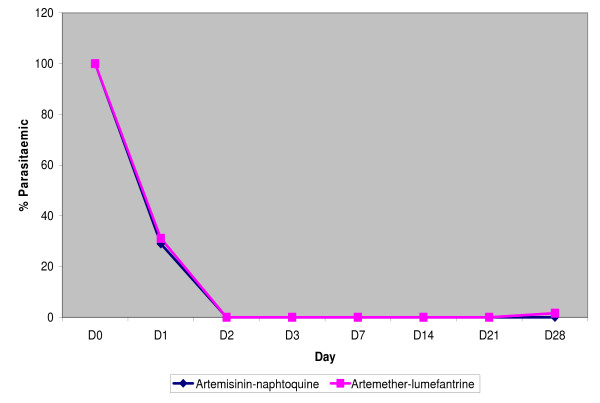
**On day 2 all slides were negative in the two treatment groups**.

**Figure 3:**
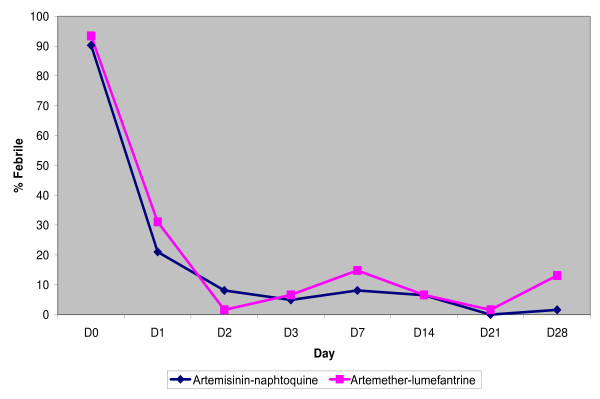
**Proportion of participants without fever (Figure 3) was similar on days 2 and 3 (p > 0.05)**.

Data of participants proportion who achieved parasites and fever clearance during the 3 days of treatment are presented in figures 2 and 3 respectively.

Day 1 slide negative results were observed for 44 (71%) of 62 AN-treated participants and 42 (68.9%) of 61 AL-treated participants. On day 2 all slides were negative in the two treatment groups (Figure 2).

Absence of fever (defined as a temperature of < 37.5°C) on day 1 was found for 49 (79%) of 62 AN participants and for 42 (68.9) of 61 AL participants.

Proportion of participants without fever (Figure 3) was similar on days 2 and 3 (p > 0.05).

### Gametocytes carriage

Gametocytes carriage was similarly low in both treatment groups. At the time of presentation, gametocytes carriage occurred in 3 of 62 participants in the AN group and 2 of AL group.

On day 7 only 3 participants (2 in the AN group and 1 in the AL group) had detectable gametocyte counts.

Among participants in whom gametocytes were found at any time during follow-up, parasite densities were < 45 parasites/ul of whole blood, and all participants had negative results of testing for gametocytes by day 28.

### Therapeutic response

Table 2 shows the results of the 28-day therapeutic efficacy of the drugs. The number of evaluable participants with adequate clinical and parasitological response (ACPR) were 62 (100%) for AN and 60 (98.4%) for AL. the difference in the cure rate of the two regimens was not statistically significant (p > 0.05).

**Table 2:** Therapeutic response by treatment group (pcr-uncorrected)

		
Classification (day 28)	AN (n = 62)	AL (n = 61)
ETF	0	0
LCF	0	1 (1.6%)
LPF	0	0
ACPR	62 (100%)	60 (98.4%)

ETF = Early treatment failure LCF = Late clinical failure LPF = Late parasitological failure ACPR = Adequate Clinical and parasitological response. As shown in Table 2, the ACPR was 100% for AN and AL on day 14 after treatment. On day 28 cure rates were 100% (62 of 62) and 98.4% (60 of 61) for participants receiving AN and AL, respectively. When the efficacy results were corrected for PCR results, an ACPR of 100% was observed with AL (61/61). This difference between treatment arms was not statistically significant (p > 0.05)

Late clinical failure was observed in one participant (1.6%) that received AL but in none of those treated with AN.

### Adverse events

The most commonly reported and possibly drug-related adverse events to both combination therapies were effects on the gastrointestinal (abdominal pain, anorexia, nausea, diarrhoea and late vomiting) and central nervous system (dizziness) (Table 3).

**Table 3:** Clinical adverse event in the two arms of the study

Adverse event	Artemisinin/naphtoquine		Artemether/lumefantrine	
	N = 62	%	N = 61	%

Asthenia	1	1.6	1	1.6

Anorexia	3	4.8	1	

Nausea	1	1.6	3	4.8

Vomitting	1	1.6	3	4.8

Diarrhoea	2	3.2	1	1.6

Dizziness	2	3.2	1	1.6

Abdominal pain	1	1.6	2	3.3

Pruritus	1	1.6	0	0

The most commonly reported and possibly drug-related adverse events to both combination therapies were effects on the gastrointestinal (abdominal pain, anorexia, nausea, diarrhoea and late vomiting) and central nervous system (dizziness)

ETF = Early treatment failure LCF = Late clinical failure LPF = Late parasitological failure ACPR = Adequate Clinical and parasitological response. As shown in Table 2, the ACPR was 100% for AN and AL on day 14 after treatment. On day 28 cure rates were 100% (62 of 62) and 98.4% (60 of 61) for participants receiving AN and AL, respectively. When the efficacy results were corrected for PCR results, an ACPR of 100% was observed with AL (61/61). This difference between treatment arms was not statistically significant (p > 0.05)

No severe alterations in renal, haematologic or hepatic function were observed with any of the drug combinations under study. The transaminase increases observed on day 7 did not exceed 2.5 × normal values. Only one participant in the AL group presented with a slight increase in creatinine levels without any serious clinical signs (Table 4).

**Table 4:** Biological tolerance to the two ACTs on days 0 and 7

	Day	Artemisinine/naphtoquine	Artemather/lumefantrine
Aneamia	0	29/60	23/60

(Hb < 11 g/d)		48.3%	38.3%

	7	36/60	34

		60%	56.7%

Craetinemia > 14	0	0/60	1/60

		0%	1.7%

	7	0/60	1/60

		0%	1.7%

SGOT > 48	0	0/60	0/60

		0%	0%

	7	0/60	0/60

		0%	0%

SGPT > 45	0	0/60	1/60

		0%	1.7%

	7	0/60	1/60

		0%	1.7%

## Discussion

Artesunate-based combinations are currently being evaluated to assess their role in developing new anti-malarial drug policies for African countries. The key goal of ACT is to enhance cure rates and delay development of parasite resistance to component drugs [16]. Côte d'Ivoire has opted for artesunate/amodiaquine (ASAQ) and AL respectively as first-line and second-line treatment of uncomplicated *falciparum *malaria, whereas other countries have primarily chosen AL. This study has shown rapid parasite and fever clearance in participants treated with six-dose regimen of AL and one day course of AN.

The combination of artemisinin and naphtoquine in the form of Arco^® ^was developed as an alternative to established combinations, such as artemether/lumefantrine. Our data show that Arco^® ^was effective and well tolerated by Ivorian children and adults with uncomplicated *falciparum *malaria. We recruited 125 participants, mostly children (58.1% in AN arm and 52.5% in AL arm), and were able to obtain validated 28-day follow-up data for 123 participants (98.4%). Recrudescence occurred within 28 days in 1 participant in artemether/lumefantrine group.

The present trial confirmed the efficacy of the six-dose regimen of AL given over three days [17]. No severe side-effects attributable to study medication were found during the follow-up period in any of the treatment groups. These data suggest that Arco could prove to be suitable for use as combination antimalarial therapy.

However, despite the promise of AN there are substantial limitations to this regimen, including one day treatment (more rapid emergence of resistance), and the lack of WHO prequalification or GMP certification.

In high transmission settings characteristic of much of sub-Saharan Africa, children who are semi-immune generally need treatment for malaria several times a year, effects of therapy on subsequent infections should also be considered. Indeed, recurrent illnesses due to recrudescence and those caused by new infections are clinically Indistinguishable [18]. Therefore, the effects of treatment on the overall risk of subsequent recurrent malaria might be regarded as the most important outcome for comparison between potential anti-malarial regimens.

This study of drug efficacy have limited follow-up to 28 days, according to WHO recommendations, and thus only the short-term effectiveness of drugs has been compared. Therefore, such study may not identify important differences between treatment regimens.

Rather, differences in effectiveness can be fully appreciated only with longer-term follow-up (42 days).

Cost, access, and local efficacy, data are also fundamental elements to consider before AN is implemented as policy. Further studies are needed to estimate the long-term safety and pharmacological action of this ACT. This will provide more information for countries where the use of this drug may be considered an option. Artemisinin derivatives in combination with classical anti-malarial drugs, which are still effective as monotherapy, represent, therefore, the best options for the treatment of malaria in situations where chloroquine resistance exists.

For malaria-control programmes to benefit fully from effective anti-malarial drugs, their regimens should be highly efficacious, of short duration, well-tolerated, and cheap. An added bonus would be the propensity to reduce transmission and limit the development of resistance, a possibility that might be achieved with the artemisinin derivatives. Governments, including Ivory Coast government, are morally obliged to subsidize the cost of treatment with ACTs to ensure the well-being of all; particularly vulnerable groups such as children under five years of age.

A major obstacle to this drug's widespread use is the lacks of WHO prequalification or GMP certification.

Further clinical trials involved large number of participants and 42 days follow-up data with pharmacokinetic studies should be conducted before its widespread use can be supported.

## Competing interests

The authors declare that they have no competing interests.

## Authors' contributions

OAT, LKP contributed to the design and coordination of the study, supervised the enrolment and follow-up of participants, assisted with data entry and interpretation, and prepared the manuscript.

BAA carried out the molecular genetic studies, participated in analysis and interpretation of data, and participated in the preparation of the manuscript.

JDY, WT, JD, GOG, OM participated in the enrolment and the follow-up of participants
